# Trends, causes, and risk factors of mortality among children under 5 in Ethiopia, 1990–2013: findings from the Global Burden of Disease Study 2013

**DOI:** 10.1186/s12963-016-0112-2

**Published:** 2016-11-14

**Authors:** Amare Deribew, Gizachew Assefa Tessema, Kebede Deribe, Yohannes Adama Melaku, Yihunie Lakew, Azmeraw T. Amare, Semaw F. Abera, Mesoud Mohammed, Abiy Hiruye, Efrem Teklay, Awoke Misganaw, Nicholas Kassebaum

**Affiliations:** 1 KEMRI-Wellcome Trust Research Programme, Kilifi, Kenya; 2 Nuffield Department of Clinical Medicine, University of Oxford, Oxford, UK; 3 St. Paul Millennium Medical College, Addis Ababa, Ethiopia; 4 School of Public Health, University of Adelaide, Adelaide, Australia; 5 Department of Reproductive Health, Institute of Public Health, University of Gondar, Gondar, Ethiopia; 6 Wellcome Trust Brighton & Sussex Centre for Global Health Research, Brighton & Sussex Medical School, Falmer, Brighton, UK; 7 School of Public Health, Addis Ababa University, Addis Ababa, Ethiopia; 8 School of Public Health, Mekelle University, Mekelle, Ethiopia; 9 School of Medicine, University of Adelaide, Adelaide, Australia; 10 Ethiopian Public Health Association, Addis Ababa, Ethiopia; 11 College of Medicine and Health Sciences, Bahir Dar University, Bahir Dar, Ethiopia; 12 Department of Epidemiology, University of Groningen, Groningen, The Netherlands; 13 Kilte Awlaelo-Health and Demographic Surveillance Site, Tigray, Ethiopia; 14 Institute of Biological Chemistry and Nutrition, Hohenheim University, Stuttgart, Germany; 15 Federal Ministry of Health, Addis Ababa, Ethiopia; 16 Institute for Health Metrics and Evaluation, University of Washington, Seattle, USA

## Abstract

**Background:**

Ethiopia has made remarkable progress in reducing child mortality over the last two decades. However, the under-5 mortality rate in Ethiopia is still higher than the under-5 mortality rates of several low- and middle-income countries (LMIC). On the other hand, the patterns and causes of child mortality have not been well investigated in Ethiopia. The objective of this study was to investigate the mortality trend, causes of death, and risk factors among children under 5 in Ethiopia during 1990–2013.

**Methods:**

We used Global Burden of Disease (GBD) 2013 data. Spatiotemporal Gaussian Process Regression (GPR) was applied to generate best estimates of child mortality with 95% uncertainty intervals (UI). Causes of death by age groups, sex, and year were measured using Cause of Death Ensemble modeling (CODEm). For estimation of HIV/AIDS mortality rate, the modified UNAIDS EPP-SPECTRUM suite model was used.

**Results:**

Between 1990 and 2013 the under-5 mortality rate declined from 203.9 deaths/1000 live births to 74.4 deaths/1000 live births with an annual rate of change of 4.6%, yielding a total reduction of 64%. Similarly, child (1–4 years), post-neonatal, and neonatal mortality rates declined by 75%, 64%, and 52%, respectively, between 1990 and 2013. Lower respiratory tract infection (LRI), diarrheal diseases, and neonatal syndromes (preterm birth complications, neonatal encephalopathy, neonatal sepsis, and other neonatal disorders) accounted for 54% of the total under-5 deaths in 2013. Under-5 mortality rates due to measles, diarrhea, malaria, protein-energy malnutrition, and iron-deficiency anemia declined by more than two-thirds between 1990 and 2013. Among the causes of under-5 deaths, neonatal syndromes such as sepsis, preterm birth complications, and birth asphyxia ranked third to fifth in 2013.

Of all risk-attributable deaths in 1990, 25% of the total under-5 deaths (112,288/435,962) and 48% (112,288/232,199) of the deaths due to diarrhea, LRI, and other common infections were attributable to childhood wasting. Similarly, 19% (43,759/229,333) of the total under-5 deaths and 45% (43,759/97,963) of the deaths due to diarrhea and LRI were attributable to wasting in 2013. Of the total diarrheal disease- and LRI-related deaths (*n* = 97,963) in 2013, 59% (57,923/97,963) of them were attributable to unsafe water supply, unsafe sanitation, household air pollution, and no handwashing with soap.

**Conclusions:**

LRI, diarrheal diseases, and neonatal syndromes remain the major causes of under-5 deaths in Ethiopia. These findings call for better-integrated newborn and child survival interventions focusing on the main risk factors.

## Background

The world has made substantial progress in reducing child mortality over the last four decades [1–4]. Several factors, such as implementation of high-impact child survival interventions, health system strengthening, improvements in maternal education and family income, commitments of policymakers and donors, and the establishment of the Millennium Development Goals (MDGs) have contributed to a reduction in child mortality globally [1].

Several countries in sub-Saharan Africa, Southern and Central Asia, and Oceania made insufficient progress to achieve MDG 4 [1, 4]. In Ethiopia, under-5 mortality has declined significantly over the last 20 years, and the country achieved the MDG related to child survival [4–8]. However, the under-5 mortality rate in Ethiopia is still higher compared to the mortality rate in many low- and middle-income countries (LMIC).

Evidence-based estimation of child mortality and the causes of child death provides a basis for planning national health strategies and tracking progress toward child survival goals [4]. However, several LMIC, including Ethiopia, have fragile health management information systems and incomplete vital registration to monitor the trends and risk factors of child mortality [9–11].

It is therefore imperative to systematically investigate the causes of child death and the risk factors to guide policymaking and child survival interventions in Ethiopia. The objective of this study was to investigate the mortality trend, causes of death, and risk factors among children under 5 in Ethiopia over the last 23 years using GBD 2013 data. The findings of this study could serve as benchmarks to track child survival goals during the Sustainable Development Goal (SDG) era and Health Sector Transformation Plan of Ethiopia.

## Methods

### Settings

Ethiopia, located in the horn of Africa, is the second-most populated country in Africa next to Nigeria, with a total population of 90 million [12]. In the last two decades, the Ethiopian health policy has focused on health promotion, disease prevention, and curative services. The country has introduced an innovative Health Extension Programme (HEP) to deliver cost-effective basic health services to all Ethiopians, mainly targeting women and children [13, 14]. Although Ethiopia has made significant progress in child mortality reduction, it has still very high under-5 (68/1000 live births), infant (44/1000 live births), and neonatal (28/1000 live births) mortality rates [13].

### Data sources and modeling

The 2013 GBD study used several sources of data such as censuses, verbal autopsy from Health and Demographic Surveillance System (HDSS) sites, Demographic and Health Surveys (DHS), and scientific literature to estimate child mortality and causes of death in Ethiopia [15].

The detailed sequential data analysis strategy to estimate the cause-specific child mortality rate is described by Wang and colleagues elsewhere [1]. Briefly, child mortality point estimates were extracted from the aforementioned data sources. First, a non-linear mixed-effects model was used to examine the relationship between child mortality with lagged distributed income per person, maternal education, and the crude death rate from HIV/AIDS in the under-5 age group. In the second stage, spatiotemporal regression was applied to the residuals in the first model to borrow strength over time and across countries in the GBD region. Results from spatiotemporal regression were then used as priors in the third stage, in which a Gaussian Process Regression (GPR) was applied to generate best estimates of child mortality with 95% uncertainty intervals. In the final stage, age and sex models were used to estimate age- and sex-specific mortality rates for neonatal, post-neonatal, child (1–4 years), and under-5 age groups [1]. The modified UNAIDS EPP-SPECTRUM suite model was used to estimate the incidence and mortality rates for HIV/AIDS [16]. Causes of death by age groups, sex, and year for other diseases were measured using CODEm. A detailed description of CODEm is reported elsewhere [16–19]. In summary, CODEm tests and selects the best-performing model from a wide range of models such as mixed-effects linear models and spatiotemporal Gaussian process regression (ST-GPR) models in predicting mortality rates and cause fractions [16]. The results of all cause-specific models are summed to ensure that the total for all specific diseases and injuries is equal to all-cause mortality.

In addition to the cause of death, we have assessed risk factors (exposures that predispose to the cause of death) for mortality. GBD 2013 used a comparative risk assessment approach to quantify the proportion of deaths attributable to the risk factors [20]. GBD classifies risk factors into three major categories: behavioral, environmental and occupational, and metabolic. Stunting, underweight, non-exclusive breastfeeding, and other dietary risk factors are included under behavioral risk factors [20]. For each risk factor, the attributable burden was estimated by comparing observed deaths to those that would have been observed if a counterfactual level of exposure had occurred in the past. For risk-outcome pairs, the attributable burden was estimated using the following equation:

$$ TA{B}_{jasct}={\displaystyle \sum_{o=1}^w}A{D}_{oasct}PA{F}_{oasct} $$

Where TAB is total attributable burden for risk factor *j* in age group *a*, sex *s*, country *c,* and year *t.* AD_oasct_ is attributable death for cause *o* in age group *a*, sex s, country *c,* and year *t*. PAF_joasct_ is the population attributable fraction (PAF) for cause *o* due to risk factor *j* in age group *a*, sex *s*, country *c,* and year *t*. The exposure level for each risk factor (p), the effect size (relative risk, RR), and the risk factor level associated with the theoretical minimum risk exposure level (TMREL) were used to calculate PAF [20].

$$ PA{F}_{asct} = \frac{{\displaystyle {\int}_{x=l}^u}R{R}_{as}(x){P}_{asct}(x)dx - R{R}_{as}(TMREL)}{{\displaystyle {\int}_{x=l}^u}R{R}_{as}(x){P}_{asct}(x)dx} $$

## Results

**Fig. 1:**
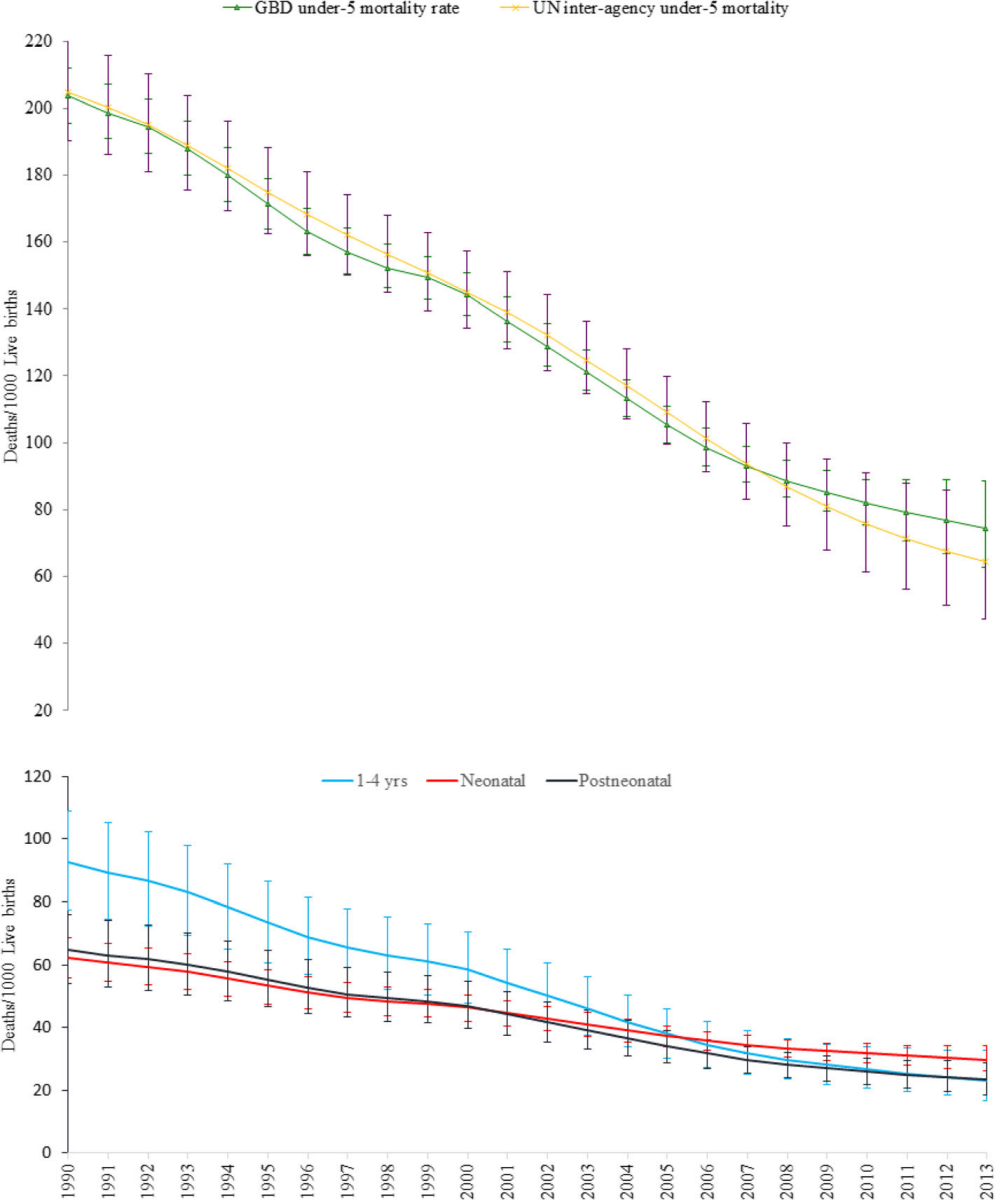
Trends of neonatal, post-neonatal, child, and under-5 mortality rates with 95% uncertainty intervals (UI) for both sexes in Ethiopia, 1990–2013

A total of 102,109 (95% UI: 83,534.5–125,027.5) girls and 127,223 (UI: 105,744.2–154,297.5) boys died in 2013 (Table 1). Neonatal and post-neonatal mortality rates fell by 52% (62.1 deaths/1000 live births to 29.9 deaths/1000 live births) and 64% (64.7 deaths/1000 live births to 23.3 deaths/1000 live births), respectively, between 1990 and 2013. In the same period, the child (1–4 years) mortality rate declined by 75% (92.5 deaths/1000 live births to 23.2 deaths/1000 live births). Between 1990 and 2013, the under-5 mortality rate declined from 203.9 deaths/1000 live births to 74.4 deaths/1000 live births with an annual rate of change of 4.6% and a total reduction of 64%. The GBD 2013 estimation of under-5 mortality was not significantly different from the UN inter-agency group estimate except from 2010 to 2013, when the GBD estimates were higher than those of the UN inter-agency group (UIAG) (Fig. 1).

**Table 1:** Number of under-5 deaths and under-5 mortality rates with 95% UI by year

Year	Number of under-5 deaths			Under-5 mortality rate		
	Males	Females	Total	Rate	95% UI	
1990	248,155	205,807	453,962	203.9	195.5	212
1995	238,598	197,896	436,494	171.3	163.8	179.1
2000	223,829	185,380	409,209	144.3	137.9	150.8
2005	169,769	139,311	309,080	105.4	99.8	110.8
2010	136,364	110,180	246,544	85.1	79.7	91.7
2013	127,223	102,109	229,333	74.4	62.7	88.4

**Fig. 2:**
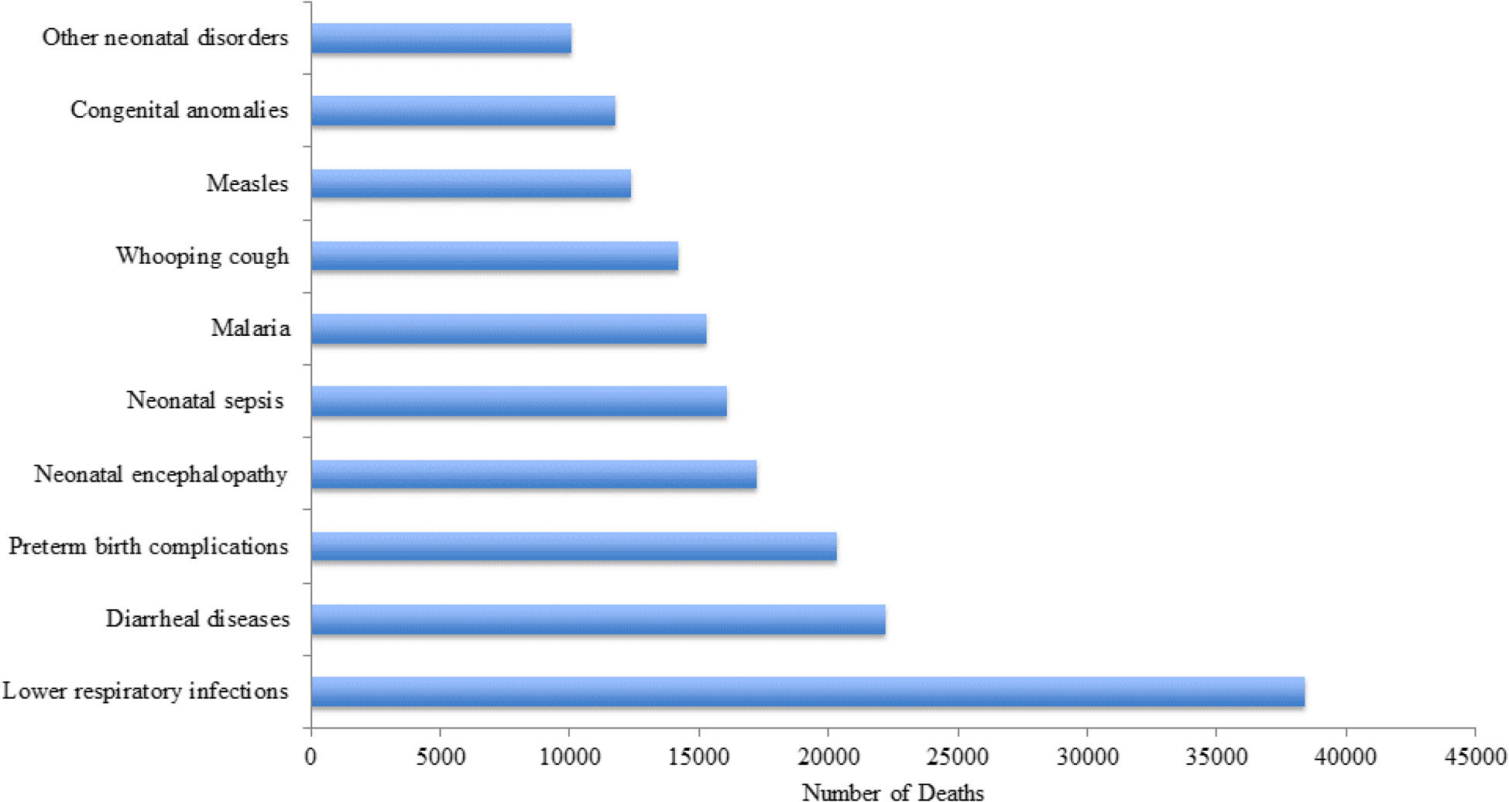
Top 10 causes of under-5 deaths in Ethiopia, 2013

Figure 2 shows the top 10 causes of under-5 deaths in 2013. Lower respiratory infections (LRI), diarrheal diseases, and neonatal syndromes (preterm birth complications, neonatal encephalopathy, neonatal sepsis, and other neonatal disorders) accounted for 54% of the total under-5 deaths.

The top 25 cause-specific under-5 mortality rates in 1990 and 2013 are described in Table 2. LRI ranked as the top cause of under-5 mortality in 1990 (747 deaths/100,000 persons) and in 2013 (271 deaths/100,000 persons). However, over the last 23 years mortality due to LRI declined by 64%. Under-5 mortality rates due to other major diseases such as measles, diarrhea, malaria, protein-energy malnutrition, and iron-deficiency anemia declined by two-thirds and more between 1990 and 2013. Neonatal syndromes such as sepsis, preterm birth complications, and birth asphyxia ranked third to fifth in 2013. Whooping cough and measles remained among the top 10 causes of death in 1990 and 2013.

**Table 2:** Cause specific under-5 mortality rates/100,000 population for both sexes in Ethiopia, 1990–2013

Cause of death or injury	1990				2013				
	Rank	Mortality rate/100,000	Lower UI	Upper UI	Rank	Mortality rate/100,000	Lower UI	Upper UI	Median %Change 1990–2013
Lower respiratory infections	1	747.13	360.99	1090.91	1	270.62	163.08	387.19	−0.64
Diarrheal diseases	3	640.25	205.52	1042.36	2	156.40	64.21	257.15	−0.76
Preterm birth complications	5	327.21	217.78	458.98	3	143.16	88.83	201.66	−0.56
Neonatal encephalopathy due to birth asphyxia and trauma	6	323.85	209.21	458.69	4	121.43	75.25	175.19	−0.63
Neonatal sepsis and other neonatal infections	9	192.10	101.28	305.83	5	113.31	65.51	186.76	−0.41
Malaria	4	435.17	147.25	827.19	6	107.58	42.88	204.27	−0.75
Whooping cough	8	213.41	0.30	1030.06	7	100.16	0.18	406.44	−0.53
Measles	2	716.98	30.15	2313.28	8	87.10	2.74	433.88	−0.88
Congenital anomalies	12	158.87	69.25	383.39	9	82.84	50.83	139.11	−0.48
Other neonatal disorders	11	165.04	98.34	280.19	10	70.93	39.21	115.49	−0.57
Protein-energy malnutrition	7	238.58	52.12	450.43	11	67.63	26.52	114.93	−0.72
Sexually transmitted diseases excluding HIV	13	11.69	49.77	218.89	12	62.99	29.68	113.23	−0.46
Meningitis	10	190.98	72.36	342.00	13	52.09	26.88	85.66	−0.73
HIV/AIDS	21	20.05	14.17	28.11	14	16.67	12.82	21.23	−0.17
Tubercolosis	16	56.45	22.15	97.05	15	12.25	6.48	19.43	−0.78
Iron-deficiency anemia	15	64.03	10.19	153.72	16	11.07	2.76	25.82	−0.83
Intestinal infectious diseases	24	11.58	2.35	22.96	17	10.15	3.29	19.56	−0.12
Road injuries	19	25.60	5.44	49.26	18.	9.45	3.45	17.50	−0.63
Drowning	20	22.71	6.41	44.23	19	8.85	3.73	20.09	−0.61
Tetanus	17	46.53	26.17	113.78	20	8.60	4.78	15.40	−0.82
Fire, Heat, and hot substances	18	26.11	5.54	52.89	21	8.60	3.42	17.24	−0.67
Exposure to mechanical forces	25	11.15	4.59	20.38	22	7.48	2.88	18.55	−0.33
Hemoglobinopathies and hemolytic anemias	22	16.68	2.23	39.55	23	5.98	2.18	11.19	−0.64
Other neoplasms	31	7.65	3.04	16.47	24	5.56	2.68	8.62	−0.27
Other infectious diseases	23	13.40	5.87	24.67	25	4.84	2.47	8.79	−0.64
Collective violence and legal intervention	14	86.87	86.21	87.56					

**Fig. 3:**
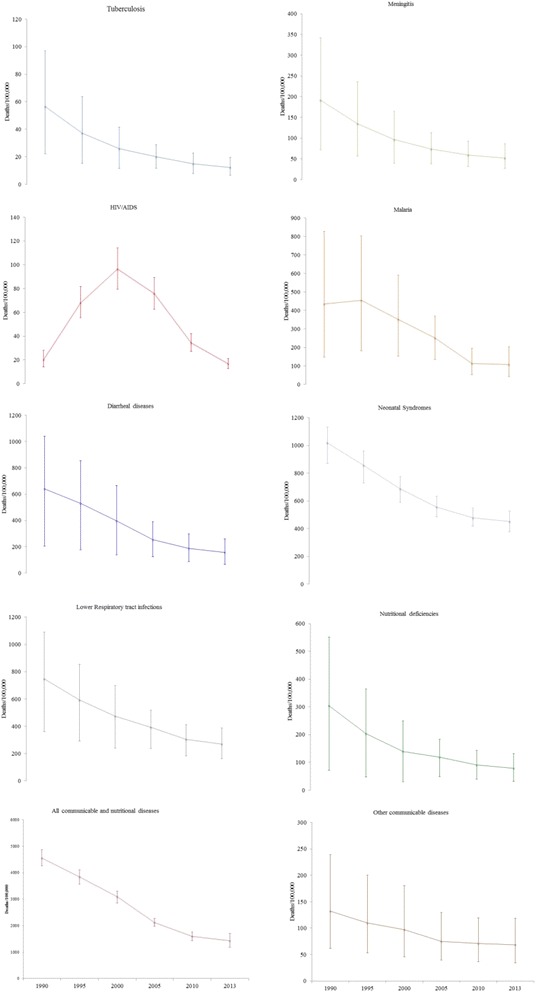
Trends of selected cause-specific under-5 mortality rates for both sexes with 95% UI

We present the trends of selected cause-specific mortality rates in Fig. 3. The trends of mortality rates due to malaria, TB, neonatal disorders, LRI, diarrhea, and nutritional disorders declined steadily between 1990 and 2013. The HIV/AIDS mortality rate showed a biphasic trend where it sharply rose until 2005 and declined steadily thereafter (Fig. 3).

**Fig. 4:**
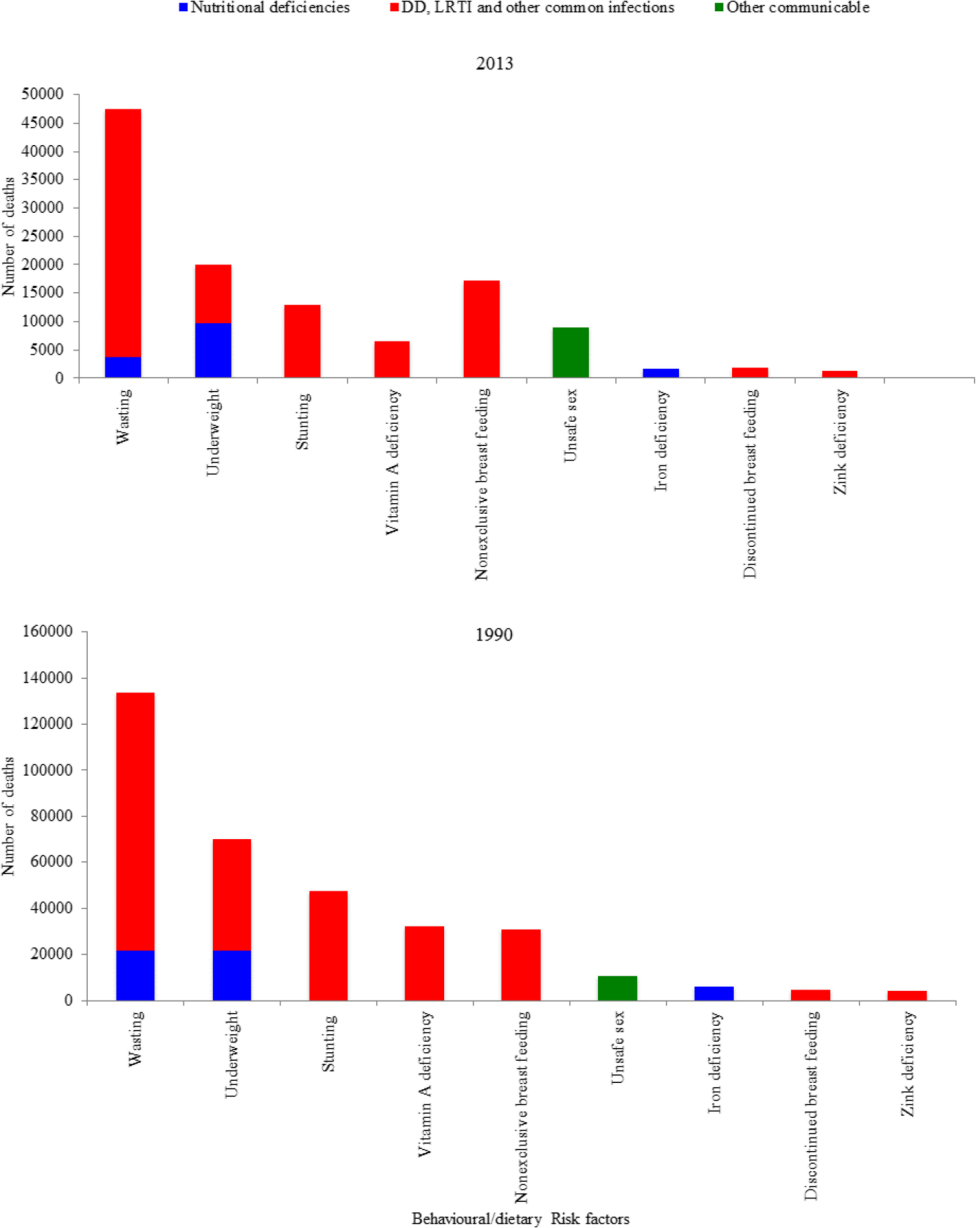
Behavioral/dietary risk factors and number of under-5 deaths, 1990–2013. DD = Diarrheal diseases, LRI = lower respiratory tract infections

Using the comparative risk assessment approach, we calculated the number of deaths attributable to the major risk factors. Childhood wasting, underweight, stunting, non-exclusive breastfeeding, and vitamin A deficiency were the major behavioral/dietary risk factors for deaths due to diarrheal diseases, LRI, and other common infections (Fig. 4). There were 232,199 deaths due to diarrhea, LRI, and other common infections in 1990, and wasting was a risk factor in almost half (112,288, 48.4%) of these deaths. Of the total under-5 deaths in 1990, 25% (112,288/453,962) were attributable to wasting. In 2013, LRI and other common infections caused 97,963 under-5 deaths, and 45% (43,759) of these deaths were attributable to wasting. Under-5 deaths due to diarrhea, LRI, and other infections that were attributable to wasting declined by 61% between 1990 and 2013. In 1990, 47,399, 32,112, and 30,941 under-5 deaths due to diarrheal diseases and LRI were attributable to stunting, vitamin A deficiency, and non-exclusive breastfeeding, respectively. However, diarrheal disease- and LRI-related deaths that were attributable to stunting, vitamin A deficiency, and non-exclusive breastfeeding fell by 73%, 80%, and 44% between 1990 and 2013 (Fig. 4).

**Fig. 5:**
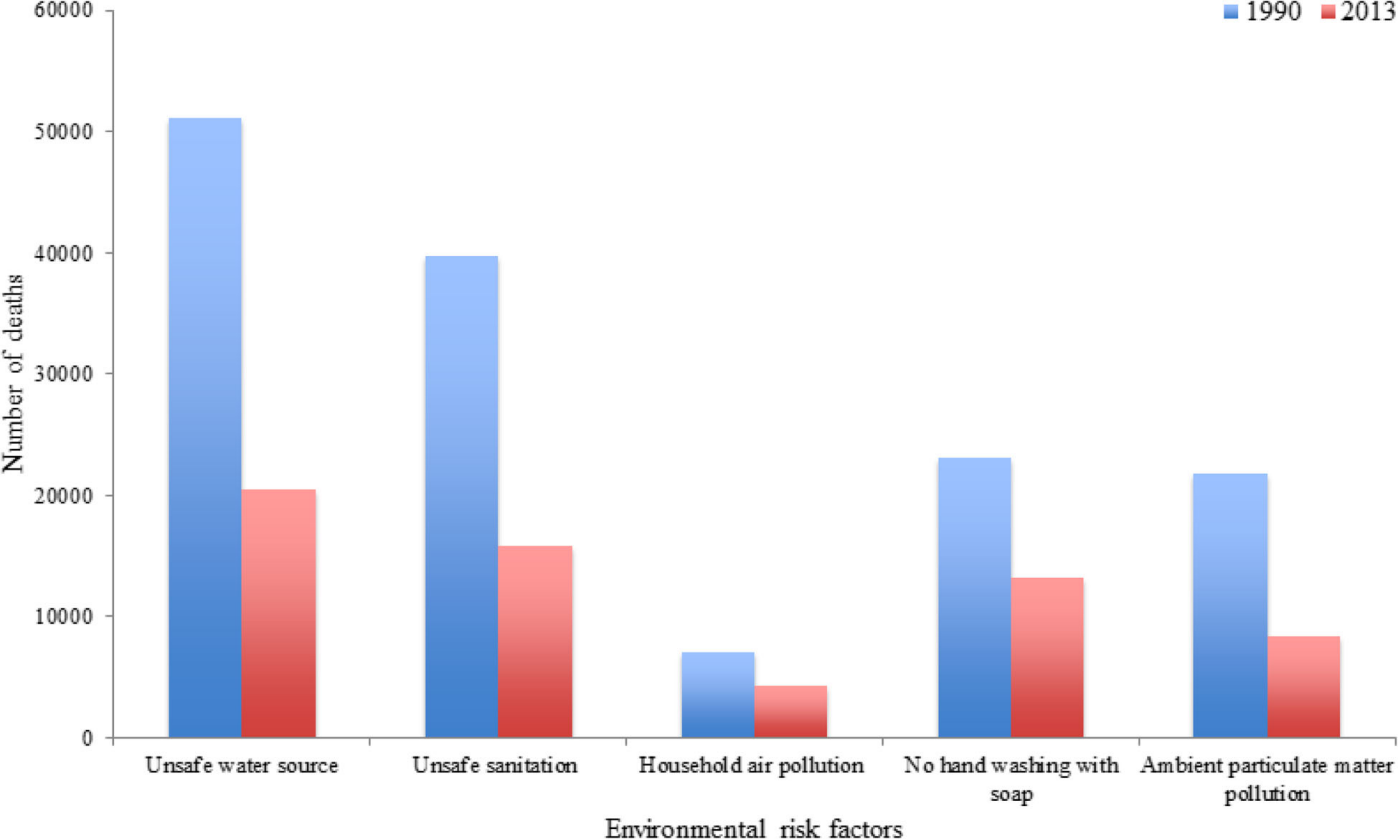
Under-5 deaths from diarrheal diseases, lower respiratory and other common infections by environmental risk factors in 1990 and 2013

In 1990, a total of 232,199 diarrheal disease- and LRI-related deaths were estimated. Of these deaths, 58% (135,816/232,199) were attributable to unsafe water supply, unsafe sanitation, household air pollution, and no handwashing with soap. Of the total diarrheal disease- and LRI-related deaths (*n* = 97,963) in 2013, 59% (57,923/97,963) were attributable to unsafe water supply, unsafe sanitation, household air pollution, and no handwashing with soap. However, deaths due to diarrheal diseases that were attributable to unsafe water supply and sanitation declined by more than 60% between 1990 and 2013 (Fig. 5).

## Discussion

According to GBD 2013, the under-5 mortality rate declined by 64% between 1990 and 2013 in Ethiopia. Child (1–4 years), post-neonatal, and neonatal mortality rates fell by 75%, 64%, and 52%, respectively, between 1990 and 2013. Lower respiratory infection, diarrheal diseases, and neonatal syndromes remained the major causes of under-5 deaths in 2013, and childhood wasting, underweight, stunting, unsafe sanitation, and unsafe water supply were the main risk factors for under-5 mortality, but all improved dramatically during the MDG era.

In spite of its rigorous and standardized methodology in estimation of child mortality and causes of death, the study has some limitations. First, cause-specific mortality data incorporated into GBD 2013 were relatively sparse (as indicated in wide 95% UI), which could lead to under- or overestimation of some of the causes of death. For instance, measles and whooping cough are included among the top 10 causes of mortality in this study, but they are not among the top 10 in the ministry of health’s (MOH) health indicator report [21]. Methodological differences between GBD and MOH could also explain the differences in the list of the top 10 causes of death. Second, the effect of high-impact interventions such as insecticide-treated nets (ITN), immunizations, and introduction of HEP were not included in the model to estimate under-5 mortality rates. Inclusion of these interventions would improve child mortality estimation in Ethiopia. Last, the GBD 2013 data for Ethiopia did not have regional or urban–rural data to show mortality variation by locations.

The UIAG estimate shows the under-5 mortality rate declined by 68% between 1990 and 2013 [8]. GBD and UIAG have comparable under-5 mortality estimation except in 2010 to 2013, where the two estimates diverge, but both indicated that Ethiopia was on track to meet MDG 4. The GBD and UIAG methods differ in many ways. GBD iterations use summary birth histories and validated birth history models to estimate under-5 mortality rates. GBD also implemented a data bias adjustment before spatiotemporal GPR for final estimation of under-5 mortality rates with 95% UI [1]. On the other hand, the UIAG pooled all data for the DHS into single data points for each survey and used traditional birth histories and other analytical techniques [8].

Ethiopia is among the few sub-Saharan African countries that have achieved the Millennium Development Goal related to child survival (MDG 4) [1]. Several factors could have contributed to the dramatic decline in under-5 mortality in Ethiopia. First, the government of Ethiopia has made health a national priority in line with the MDG declaration and has invested massively in health infrastructure and training of health professionals over the last 20 years [13, 22]. Second, tailored community-based child survival interventions such as immunization and community-based management for major diseases such as malaria, pneumonia, and diarrheal diseases have been conducted through the flagship of HEP over the last 10 years [13, 14, 23]. The HEP involves trained and salaried female health extension workers who provide basic primary health care services at the community level. The HEP has improved maternal and newborn health care practices and could contribute to the reduction of under-5 mortality [24, 25]. The contribution of development partners to support high-effect child survival interventions such as immunization and malaria control program could also contribute significantly to reduction of under-5 mortality. For instance, malaria incident cases and deaths in Ethiopia substantially declined after the introduction of artemisinin combination therapy (ACT) and insecticide-treated nets (ITN) [26]. Improved coverage of immunization has also contributed to the dramatic decline in the measles mortality rate (>75%) over the last 23 years. The coverage of other childhood immunizations in Ethiopia such as pentavalent, pneumococcal conjugate, and rotavirus vaccines has also improved recently [13]. Last, rapid socioeconomic development in the last 10 years and decreased war and violence in Ethiopia over the last 25 years could have had significant impact on child survival [13].

Ethiopia should address several environmental, behavioral, and health system bottlenecks to achieve the ongoing child survival goals during the SDG era. Reaching the SDG under-5 mortality target of less than 25 deaths per 1000 will require continued improvement that is faster than the rate of improvement during the MDG era [27]. Doing so will require targeting behavioral risk factors such as unsafe sanitation, unsafe water supply, and household air pollution to reduce or hopefully eliminate deaths due to diarrheal diseases and LRI. On the other hand, other risk factors for child mortality, such as maternal illiteracy and poor practices, [28–30] will be addressed through behavioral change communication by the HEP. With the reduction of infectious diseases, neonatal syndromes have emerged as the major cause of child death in Ethiopia. This could be due to the low coverage (16%) of institutional delivery [13] and several health system bottlenecks such as poor quality of care and lack of well-trained health workforce at the peripheral health facilities. Integrated newborn interventions [31–33] such as home-based newborn care, high coverage of institutional delivery, and improved management of neonatal syndromes in all health facilities through trained health workers could help Ethiopia achieve the neonatal mortality target during the SDG era. Malnutrition as a cause of death and risk factor for other diseases is still a major health problem in Ethiopia. Several factors that are linked to malnutrition such as food insecurity and poor infant and child feeding practices [34] need to be addressed through multisectoral approaches by involving the community and other sectors such as education and agriculture.

## Conclusions

Ethiopia achieved MDG 4 by 2013. However, LRI, diarrheal diseases, and neonatal syndromes still remain the major causes of under-5 deaths in Ethiopia. Ethiopia should strengthen integrated newborn and child survival interventions during the SDG era. Multisectoral interventions should be developed to address the behavioral/dietary and environmental risk factors.
